# IC2Bert: masked gene expression pretraining and supervised fine tuning for robust immune checkpoint blockade (ICB) response prediction

**DOI:** 10.1038/s41598-025-14166-x

**Published:** 2025-08-01

**Authors:** Seongyong Park, Seonkyu Kim, Peng Jiang

**Affiliations:** 1 Cancer Data Science Lab, CCR, NCI, NIH, Bethesda, MD 20892 USA; 2 Aging Convergence Research Center, KRIBB, Daejeon, 34141 Republic of Korea

**Keywords:** Immune checkpoint blockade, Bulk RNAseq, Self-supervised pretraining, Supervised fine-tuning, Leave-one-dataset-out cross-validation (LODOCV), Cancer immunotherapy, Machine learning, Predictive medicine

## Abstract

Bulk RNA-seq-based prediction of immune checkpoint blockade (ICB) responses has been extensively studied to distinguish responders from non-responders. However, cohort heterogeneity remains a major challenge, hindering the robustness and generalizability of predictive models across diverse RNA-seq datasets. In this study, we present IC2Bert, a novel model that employs masked gene expression pretraining combined with domain-specific supervised fine-tuning to enhance predictive robustness across heterogeneous ICB response cohorts. To ensure an objective evaluation, we assessed the model’s performance using a Leave-One-Dataset-Out Cross-Validation (LODOCV) approach. IC2Bert demonstrated significantly improved predictive accuracy and robustness compared to existing methods, effectively addressing the challenges posed by cohort heterogeneity. The IC2Bert model and its source code are publicly available on GitHub: https://github.com/data2intelligence/ic2bert.

## Introduction

Immune checkpoint blockade (ICB) therapies have transformed cancer treatment by enabling the immune system to recognize and attack tumor cells^1^. Agents targeting immune checkpoints such as PD-1, PD-L1, and CTLA-4 have achieved remarkable success in treating various malignancies, including melanoma^2^, non-small cell lung cancer^3^, and renal cell carcinoma^4^. Nonetheless, clinical response to ICB remain highly variable, with only a fraction of patients experiencing substantial benefits^5,6^. Predicting which patients will respond to ICB remains a central challenge in oncology, underscoring the need for reliable predictive biomarkers and models. Bulk RNA sequencing (RNA-seq) has played a key role in identifying gene expression signatures that distinguish responders from non-responders to ICB therapies. For example, the Tumor Inflammation Signature (TIS) predicts responses to pembrolizumab in multiple solid tumors^7^, while the Cytolytic Activity Score (CYT), based on the expression of specific immune-related genes, correlates with improved survival in colorectal cancer^8^. Additionally, frameworks like Tumor Immune Dysfunction and Exclusion (TIDE) evaluate T-cell activity from RNA-seq data, aiding in patient stratification for ICB therapies^9^. These advancements highlight the promise of bulk RNA-seq for generating biomarkers that advance personalized cancer treatments. Despite these advances, predictive biomarkers often lose effectiveness when extended to diverse patient populations. Differences in demographics, tumor types, sequencing platforms, and data processing methods contribute to cohort heterogeneity, posing significant challenges to the generalizability of predictive models. For instance, PD-L1 expression was predictive in only 28.9% of cases, influenced by tissue types, cutoffs and cell-based assessments^10^. Similarly, tumor mutational burden (TMB) shows inconsistent predictive power across cancer types, being effective in melanoma and non-small cell lung cancer but less so in colorectal cancer^11^. Moreover, Bareche et al. demonstrated that just over half of curated gene expression signatures (22 out of 37; 59%) maintained significant associations with pan-cancer immunotherapy response^12^, emphasizing the need for careful data integration, refined modeling strategies, and rigorous validation. Machine learning (ML) and deep learning (DL) methods have emerged as potent tools for improving ICB response prediction. Leveraging diverse data sources–including transcriptomics^13^, clinical records^14^, and imaging^15^–these methods have identified novel biomarkers and achieved enhanced generalizability^16^. For example, ML models can incorporate features such as tumor mutational burden (TMB)^17^, microsatellite instability (MSI)^18^, neoantigen profiles^19^, immune cell infiltration^20^ and gut microbiota compositions^21^. Integrative approaches combining multiple data modalities address the limitations of single biomarkers, ultimately offering a more comprehensive understanding of patient responses. However, ML- and DL-based approaches for ICB response prediction still face challenges related to cohort heterogeneity, overfitting, and the reliance on large, standardized training and validation datasets. Harmonizing data across cohorts and validating models in independent clinical trials are critical but remain difficult to achieve. Recent advances in self-supervised pretraining and transfer learning offer promising strategies to address these challenges. Self-supervised pretraining allows models to learn generalized patterns from unlabeled data, while transfer learning adapts these representations to specific tasks, improving predictive accuracy and bolstering robustness against clinical and genomic variability. In this study, we present IC2Bert, a novel computational framework designed to improve ICB response prediction across heterogeneous RNA-seq cohorts. By employing masked gene expression pretraining, IC2Bert captures nuanced, dataset-agnostic gene relationships through the reconstruction of randomly masked data. To further enhance generalizability and specificity of the model, the model incorporates domain-specific supervised transfer learning, fine-tuning the pretrained representations on labeled, cohort-specific datasets while preserving broad, generalized knowledge. Using a Leave-One-Dataset-Out Cross-Validation (LODOCV) strategy–simulating real-world scenarios with unseen data–IC2Bert achieved substantially higher predictive accuracy and robustness than existing approaches, effectively addressing the challenges posed by cohort heterogeneity. By combining masked gene expression pretraining with transfer learning, IC2Bert represents a significant advancement in predictive modeling for immunotherapy. It provides a promising tool for accurately predicting patient responses to ICB therapy using only bulk RNA-seq profiles. This capability may also facilitate the integration of models from other modalities, forming comprehensive, multimodal frameworks that further enhance personalized treatment planning and improve clinical outcomes. To support ongoing research and development, we have made IC2Bert and its source code publicly available on GitHub: https://github.com/data2intelligence/ic2bert.

## Results

### Overall training and evaluation strategy

**Fig. 1:**
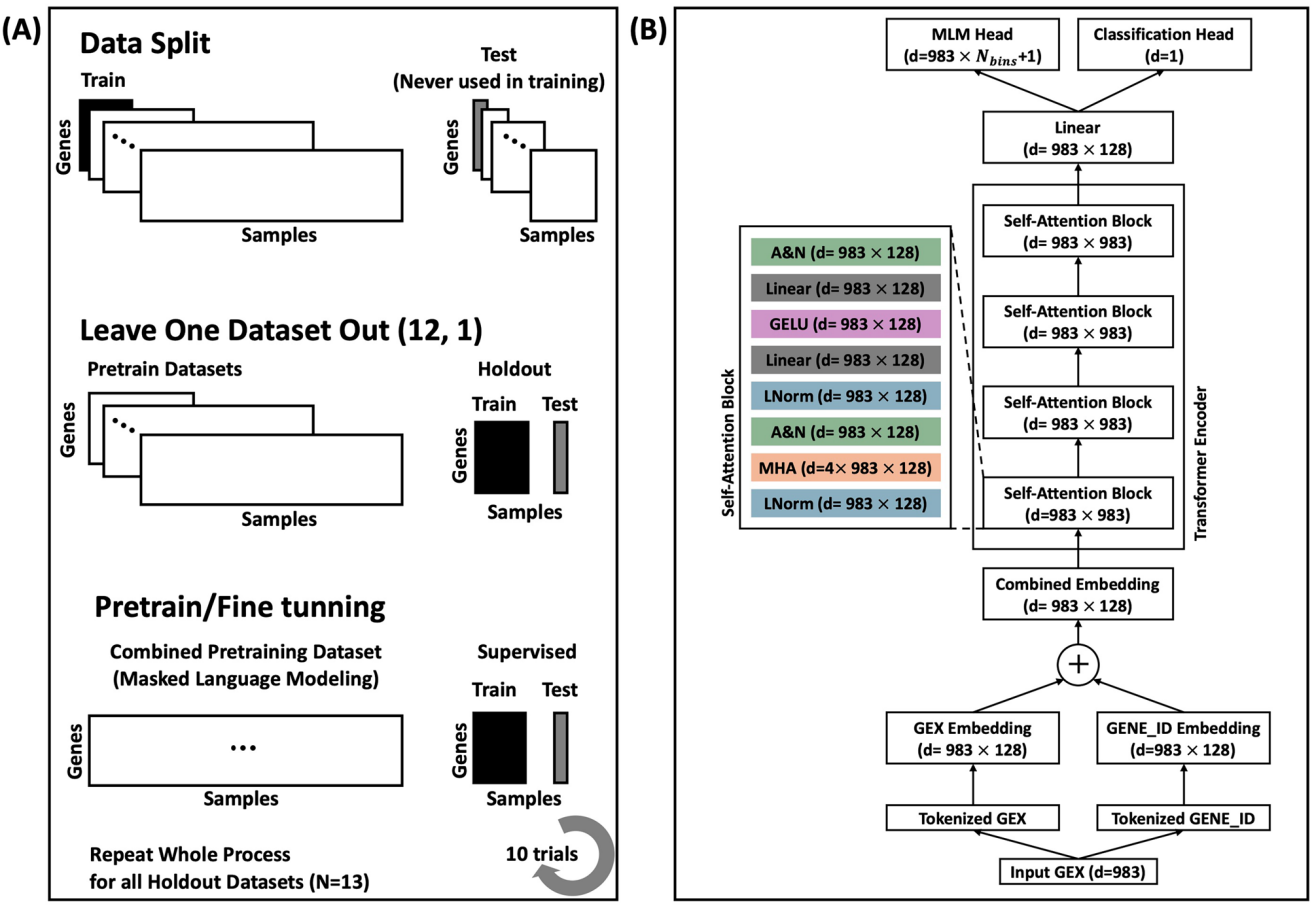
(**A**) Training and Validation (Iterative LODOCV) Scheme for IC2Bert. (**B**) Model Architecture of IC2Bert.

We employed an iterative Leave-One-Dataset-Out Cross-Validation (LODOCV) framework (Fig. 1) to thoroughly evaluate IC2Bert’s capacity for generalization across diverse datasets. In each iteration, one dataset served as the holdout set, while the remaining 12 were used for pretraining. Although the holdout dataset remained entirely excluded during pretraining, we subsequently applied transfer learning on its training subset to tailor the model to the target domain before evaluating performance on its test subset. This procedure was repeated for all 13 datasets, ensuring that each one served as the holdout dataset in turn.

Each dataset was split into a training (80%) and test (20%) subset using stratified sampling. These internal splits serve distinct roles. During pretraining, IC2Bert learned generalized gene expression patterns through masked language modeling, using only the training subsets of the 12 pretraining datasets. The test subsets of these 12 datasets were completely held out. Most critically, the test subset of the holdout dataset was never used at any stage of the process–not during pretraining, not during transfer learning, and not for hyperparameter selection–and was reserved strictly for final performance evaluation. Following pretraining, we fine-tuned IC2Bert on the training subset of the holdout dataset and assessed its performance on the corresponding test subset. Each scenario was repeated $N=10$ times with different training/test splits. We quantified classification performance using the Area Under the Receiver Operating Characteristic Curve (AUROC).

To further clarify the influence of transfer learning, we compared zero-shot predictions (no fine-tuning on the target dataset) and transfer learning results. We also evaluated an ensemble approach, where predictions from 12 fine-tuned models (each pretrained on different datasets and fine-tuned on the target’s training subset) were combined to generate predictions on the holdout test subset. In addition, we conducted sample size analyses (discussed later) to determine how varying the number of training samples affected transfer learning outcomes. For completeness, we performed transfer learning on the 12 training datasets themselves, examining how effectively the model adapted to domains included in the pretraining phase (Table 1).

**Table 1:** Patient statistics of ICB response cohorts.

Dataset	Year	ICB Type	Drug	$N$ $^a$	$N_{\textrm{train}}$ $^b$	$N_{\textrm{test}}$ $^c$
Kim2018^44^	2018	aPD1	Pembrolizumab	45	36	9
Atezo_Finn2020^45^	2020	aPDL1	Atezolizumab	43	34	9
Yang2021^46^	2021	aPD1	Pembrolizumab	64	51	13
Atezo+Bev_McDermott2018^47^	2018	aPDL1+aVEGF	Atezolizumab+Bevacizumab	82	66	16
Atezo_McDermott2018^47^	2018	aPDL1	Atezolizumab	74	59	15
Mariathasan2018^48^	2018	aPDL1	Atezolizumab	298	238	60
Atezo+Bev_Finn2020^45^	2020	aPDL1+aVEGF	Atezolizumab+Bevacizumab	245	196	49
VanAllen2015^49^	2015	aCTLA4	Ipilimumab	42	34	8
Liu2019^50^	2019	aPD1	Pembrolizumab/Nivolumab	121	97	24
Hugo2016^51^	2016	aPD1	Pembrolizumab/Nivolumab	26	21	5
Riaz2017^52^	2017	aPD1	Nivolumab	51	41	10
Ravi2023^53^	2023	aPD1	Pembrolizumab/Nivolumab	90	72	18
Miao2018^54^	2018	aPD1	Nivolumab	33	26	7
Total				1,214	971	243

$^a$Total number of samples. $^b$Number of samples used for training. $^c$Number of samples reserved for testing.

### Ablation study for number of bins ($N_{bins}$) parameter of tokenizer

**Fig. 2:**
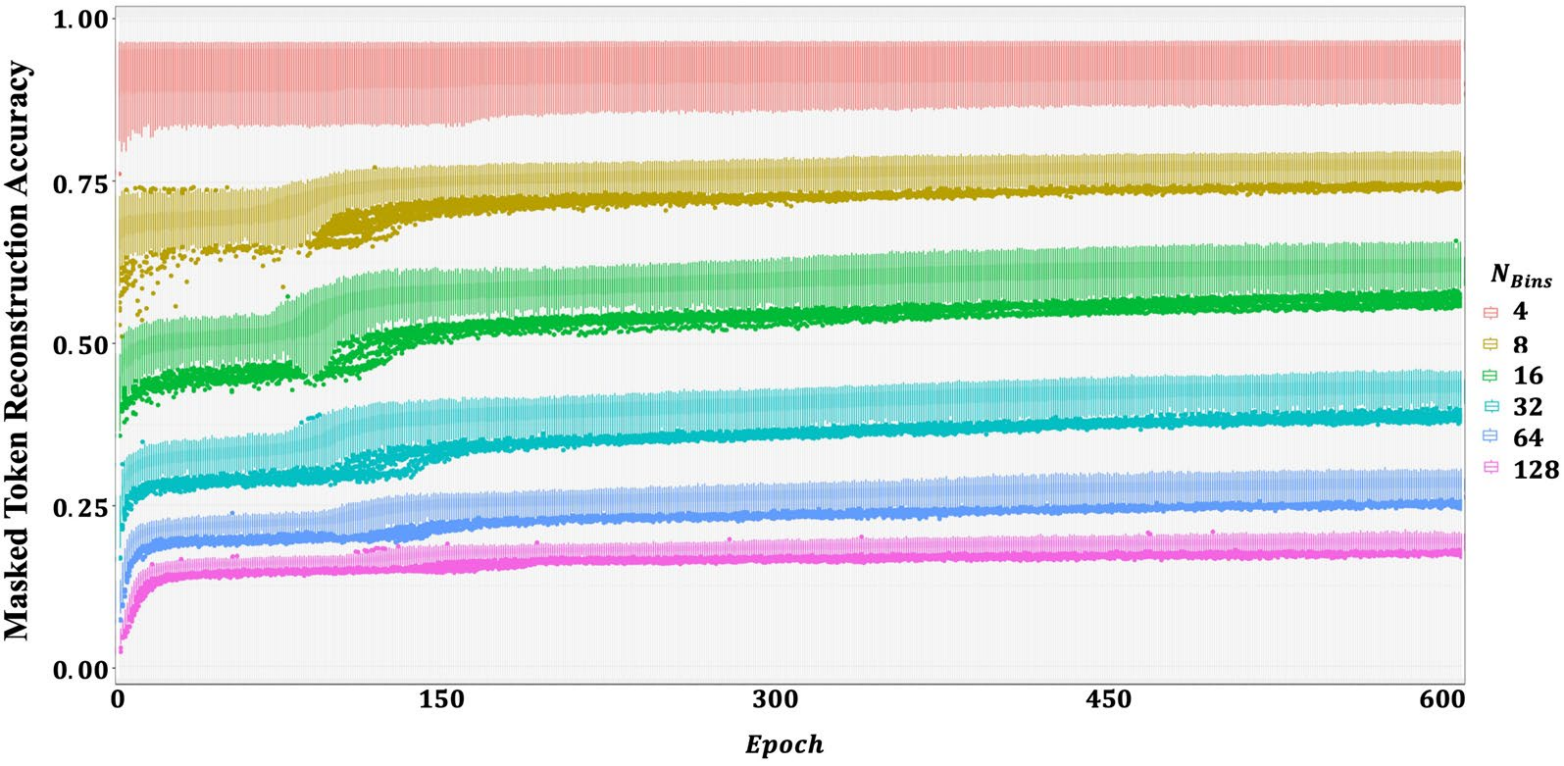
Masked token reconstruction accuracy (validation) by number of bins ($N_{bins}$).

**Fig. 3:**
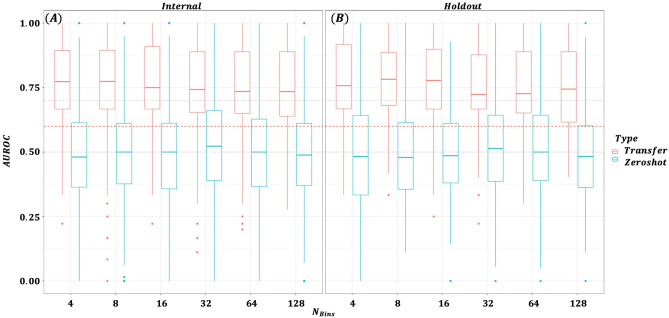
Transfer learning versus zero-shot prediction AUROC by $N_{bins}$.

**Fig. 4:**
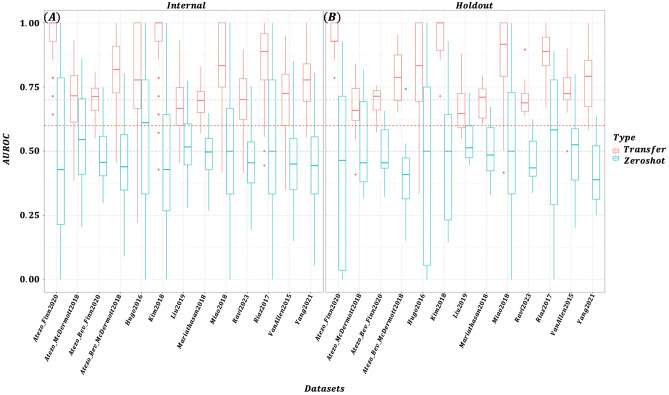
TL versus zero-shot prediction AUC by ICB datasets ($N_{bins}=4$).

We conducted an ablation study to determine how varying the number of bins ($N_{bins}$) in the gene expression tokenizer affects both masked token reconstruction accuracy during pretraining and downstream classification performance. Figure 2 illustrates the impact of different bin sizes on pretraining masked token reconstruction accuracy. With four bins, the model maintained approximately 95% accuracy throughout training, whereas at 128 bins, accuracy remained around 20% by epoch 600. This outcome is intuitive–fewer bins simplify the prediction task, reducing representational complexity and making it easier for the model to accurately recover masked tokens. Figure 3 compares AUROC performance for transfer learning and zero-shot prediction at various bin sizes. Panel (A) displays results for datasets included in the pretraining phase, while Panel (B) shows outcomes for holdout datasets. Models pretrained with fewer bins generally exhibited better transfer learning performance. In contrast, zero-shot predictions were poor regardless of bin count, though a slight improvement was noted with larger bin sizes. The performance differences between pretraining and holdout datasets were negligible. As shown in Table 2, although $N_{bins}=4$ achieved the highest mean performance (0.781), results with 8 and 16 bins were comparable (0.775 and 0.774, respectively). Figure 4 presents a dataset-level comparison of AUROC for both transfer learning and zero-shot prediction. Panel (A) reports performance on pretraining datasets, while Panel (B) focuses on holdout datasets. As summarized in Table 3, transfer learning yielded AUROCs ranging from 0.668 to 0.943 (mean 0.781), whereas zero-shot predictions ranged from 0.420 to 0.585 (mean 0.518). Ensemble predictions–derived from 12 models fine-tuned on each pretraining dataset–improved slightly over zero-shot predictions, with AUROCs ranging from 0.493 to 0.807 (mean 0.593). Since the MLP model trained and tested on the same dataset produced AUROCs of 0.500 to 1.000 (mean 0.611), the transfer learning approach on the target dataset alone showed an overall performance enhancement. Collectively, these results confirm that pretraining, followed by targeted domain-specific transfer learning, significantly improves predictive accuracy and enables the model to better adapt to the unique characteristics of each dataset.

**Table 2:** Dataset-specific ICB response prediction AUC according to $N_{bins}$ (mean ± std).

Datasets	$N_{bins}$					
	4	8	16	32	64	128
Kim2018	0.943±0.10	0.929±0.14	0.957±0.08	0.929±0.11	0.971±0.05	0.971±0.04
Atezo_Finn2020	0.936±0.07	0.929±0.11	0.950±0.07	0.950±0.08	0.964±0.05	0.921±0.08
Yang2021	0.781±0.14	0.775±0.10	0.744±0.14	0.767±0.13	0.656±0.16	0.661±0.12
Atezo+Bev_McDermott2018	0.791±0.11	0.764±0.10	0.765±0.13	0.755±0.11	0.753±0.17	0.750±0.14
Atezo_McDermott2018	0.668±0.13	0.712±0.13	0.669±0.16	0.687±0.17	0.707±0.17	0.652±0.17
Mariathasan2018	0.697±0.07	0.677±0.07	0.667±0.05	0.675±0.06	0.672±0.06	0.673±0.04
Atezo+Bev_Finn2020	0.690±0.06	0.750±0.08	0.724±0.05	0.736±0.07	0.727±0.05	0.742±0.07
VanAllen2015	0.735±0.12	0.825±0.11	0.885±0.09	0.710±0.14	0.700±0.19	0.750±0.15
Liu2019	0.677±0.12	0.677±0.10	0.657±0.09	0.683±0.07	0.696±0.06	0.641±0.07
Hugo2016	0.811±0.22	0.733±0.19	0.700±0.21	0.678±0.24	0.689±0.17	0.733±0.20
Riaz2017	0.872±0.10	0.852±0.15	0.938±0.06	0.839±0.23	0.867±0.15	0.861±0.21
Ravi2023	0.709±0.08	0.730±0.12	0.725±0.13	0.684±0.07	0.665±0.10	0.662±0.14
Miao2018	0.842±0.22	0.725±0.23	0.683±0.25	0.733±0.21	0.742±0.21	0.725±0.17
Total	0.781±0.15	0.775±0.15	0.774±0.17	0.756±0.16	0.755±0.17	0.750±0.16

$N_{bins}$: The number of bins in gene expression tokenizer.

**Table 3:** Comparison between single-dataset train/test and IC2Bert strategy (mean±std).

Datasets	MLP$^a$	IC2Bert		
		Zero-shot$^b$	Ensemble$^c$	Transfer Learn$^d$
Kim2018	1.000±0.18	0.577±0.21	0.807±0.22	0.943±0.10
Atezo_Finn2020	0.714±0.19	0.585±0.30	0.493±0.30	0.936±0.07
Yang2021	0.611±0.17	0.528±0.14	0.572±0.16	0.781±0.14
Atezo+Bev_McDermott2018	0.635±0.08	0.528±0.17	0.606±0.24	0.791±0.11
Atezo_McDermott2018	0.625±0.17	0.531±0.15	0.581±0.20	0.668±0.13
Mariathasan2018	0.632±0.08	0.543±0.08	0.589±0.10	0.697±0.07
Atezo+Bev_Finn2020	0.635±0.08	0.554±0.12	0.594±0.08	0.690±0.06
VanAllen2015	0.750±0.18	0.420±0.17	0.600±0.20	0.735±0.12
Liu2019	0.667±0.19	0.486±0.15	0.505±0.17	0.677±0.12
Hugo2016	0.444±0.22	0.515±0.27	0.611±0.22	0.811±0.22
Riaz2017	0.565±0.13	0.520±0.30	0.586±0.28	0.872±0.10
Ravi2023	0.500±0.23	0.436±0.14	0.625±0.09	0.709±0.08
Miao2018	1.000±0.18	0.511±0.20	0.542±0.27	0.842±0.22
Total	0.611±0.17	0.518±0.20	0.593±0.21	0.781±0.15

$^a$ Train and test multi-layer perceptron (MLP) on single dataset. $^b$ Zero-shot prediction performance of IC2Bert without fine-tuning. $^c$ Ensemble performance using fine-tuned IC2Bert models on each holdout dataset. $^d$ Transfer-learning performance of IC2Bert on the holdout dataset’s training subset.

### Performance comparison with other strategies

We evaluated two alternative strategies for predicting ICB response from bulk RNA-seq datasets and compared their performance to that of IC2Bert on the same test sets. The first approach employed gene set–based predictors derived from previously published literature^12^. Such methods typically use averages of expression values from predefined gene markers or apply sample-dependent averaging techniques like single-sample gene set enrichment analysis (ssGSEA)^22^. We tested 34 curated gene sets (Supplementary Table S1) from a recent publication, identifying two signatures–Cytotoxic T cell and TGFB–that performed best among the curated predictors, and we also evaluated a composite score combining both signatures. As shown in Table 4, IC2Bert’s pretraining and transfer-learning strategy substantially outperformed these gene set–based methods, achieving roughly a 28% improvement across datasets. Notably, on the small-sample Miao2018 cohort, IC2Bert reached an AUROC of $0.943 \pm 0.100$, versus $0.450 \pm 0.191$ for the top gene set predictor.

Next, we compared IC2Bert against three baseline neural architectures–DNN, 1D CNN and the Scale-Invariant Neural network Classifier (SINC)^23^–as well as six state-of-the-art domain-generalization neural network (DGNN) models: Domain Adversarial Neural Network (DANN)^24^, Invariant Risk Minimization (IRM)^25^, MetaReg^26^, MMD-AAE^27^, CrossGrad^28^ and LFR^29^. DGNNs are designed to learn domain-invariant features and predict in a new domain without additional fine-tuning. As summarized in Table 5, the baseline models delivered only moderate cross-cohort performance (e.g., on Miao2018: DNN $0.521 \pm 0.228$, CNN $0.450 \pm 0.191$, SINC $0.500 \pm 0.197$), and the DGNN approaches offered only marginal gains over these baselines (e.g., DANN $0.504 \pm 0.233$ on Miao2018) while still struggling on small-sample or distribution-shift cohorts (Table 6). In contrast, IC2Bert’s domain-specific fine-tuning consistently mitigated performance degradation under these challenging conditions–achieving $0.943 \pm 0.100$ on Miao2018 and delivering superior AUROCs across every test set–underscoring the critical importance of explicit target-domain adaptation for robust and generalizable ICB response prediction.

**Table 4:** Comparison with gene set–based predictors (mean±std).

Datasets	$GS_{Tcell}$ $^a$	$GS_{TGF\beta }$ $^b$	Composite ($GS_{Tcell}+GS_{TGF\beta }$)	IC2Bert
Kim2018	0.677±0.07	0.491±0.08	0.685±0.07	0.943±0.10
Atezo_Finn2020	0.792±0.18	0.439±0.21	0.791±0.17	0.936±0.07
Yang2021	0.604±0.15	0.537±0.18	0.612±0.16	0.781±0.14
Atezo+Bev_McDermott2018	0.759±0.11	0.493±0.12	0.708±0.12	0.791±0.11
Atezo_McDermott2018	0.604±0.15	0.584±0.14	0.609±0.15	0.668±0.13
Mariathasan2018	0.563±0.08	0.558±0.08	0.597±0.07	0.697±0.07
Atezo+Bev_Finn2020	0.677±0.07	0.491±0.08	0.685±0.07	0.690±0.06
VanAllen2015	0.706±0.18	0.554±0.18	0.723±0.16	0.735±0.12
Liu2019	0.558±0.10	0.486±0.11	0.537±0.10	0.677±0.12
Hugo2016	0.428±0.24	0.817±0.16	0.627±0.24	0.811±0.22
Riaz2017	0.650±0.23	0.518±0.24	0.650±0.21	0.872±0.10
Ravi2023	0.564±0.14	0.581±0.12	0.643±0.13	0.709±0.08
Miao2018	0.512±0.21	0.402±0.22	0.450±0.23	0.842±0.22
Total	0.630±0.19	0.551±0.20	0.649±0.19	0.781±0.15

$^a$Cytotoxic CD8 T-cell signature from Jiang et al.^9^. $^b$TGF-$\beta$ signature from Mariathasan et al.^48^.

**Table 5:** Comparison of baseline architectures and IC2Bert strategy (mean ± std).

Datasets	DNN	CNN	SINC	IC2Bert
Kim2018	0.814±0.16	0.671±0.32	0.786±0.22	0.943±0.10
Atezo_Finn2020	0.764±0.18	0.729±0.19	0.724±0.21	0.936±0.07
Yang2021	0.675±0.16	0.733±0.12	0.659±0.18	0.781±0.14
Atezo+Bev_McDermott2018	0.686±0.11	0.692±0.10	0.678±0.12	0.791±0.11
Atezo_McDermott2018	0.666±0.15	0.670±0.12	0.624±0.15	0.668±0.13
Mariathasan2018	0.634±0.06	0.573±0.05	0.642±0.08	0.697±0.07
Atezo+Bev_Finn2020	0.616±0.08	0.585±0.08	0.645±0.08	0.690±0.06
VanAllen2015	0.590±0.19	0.665±0.20	0.543±0.20	0.735±0.12
Liu2019	0.558±0.09	0.547±0.12	0.539±0.10	0.677±0.12
Hugo2016	0.456±0.23	0.556±0.24	0.536±0.24	0.811±0.22
Riaz2017	0.575±0.22	0.611±0.23	0.629±0.17	0.872±0.10
Ravi2023	0.527±0.10	0.514±0.13	0.590±0.14	0.709±0.08
Miao2018	0.521±0.23	0.450±0.19	0.500±0.24	0.842±0.22
**Total**	0.630±0.18	0.596±0.19	0.623±0.19	0.781±0.15

DNN (Deep Neural Network), CNN (1D Convolutional Neural Network), SINC (Scale-Invariant Neural network Classifier)^23^, IC2Bert (Transformer-based ICB response prediction model with domain-specific fine-tuning).

**Table 6:** Comparison with domain generalization methods and IC2Bert strategy (mean±std).

Datasets	DANN	IRM	MetaReg	MMD-AAE	CrossGrad	LFR	IC2Bert
Kim2018	0.841±0.18	0.832±0.19	0.831±0.18	0.832±0.18	0.845±0.17	0.826±0.16	0.943±0.10
Atezo_Finn2020	0.621±0.24	0.599±0.25	0.604±0.25	0.621±0.24	0.573±0.22	0.611±0.23	0.936±0.07
Yang2021	0.681±0.16	0.672±0.17	0.672±0.17	0.678±0.16	0.678±0.16	0.679±0.15	0.781±0.14
Atezo+Bev_McDermott2018	0.680±0.12	0.675±0.12	0.676±0.12	0.676±0.13	0.674±0.13	0.668±0.12	0.791±0.11
Atezo_McDermott2018	0.621±0.15	0.620±0.15	0.620±0.15	0.622±0.16	0.615±0.15	0.634±0.15	0.668±0.13
Mariathasan2018	0.635±0.08	0.621±0.09	0.620±0.09	0.619±0.08	0.624±0.08	0.628±0.07	0.697±0.07
Atezo+Bev_Finn2020	0.615±0.09	0.618±0.09	0.620±0.09	0.609±0.09	0.622±0.09	0.626±0.08	0.690±0.06
VanAllen2015	0.669±0.17	0.658±0.18	0.658±0.17	0.655±0.18	0.676±0.18	0.666±0.18	0.735±0.12
Liu2019	0.559±0.11	0.562±0.11	0.565±0.11	0.576±0.11	0.565±0.12	0.558±0.12	0.677±0.12
Hugo2016	0.486±0.24	0.512±0.25	0.515±0.24	0.513±0.24	0.483±0.22	0.528±0.25	0.811±0.22
Riaz2017	0.545±0.19	0.569±0.19	0.568±0.19	0.558±0.20	0.576±0.22	0.572±0.19	0.872±0.10
Ravi2023	0.596±0.12	0.564±0.13	0.563±0.12	0.567±0.13	0.570±0.13	0.573±0.12	0.709±0.08
Miao2018	0.504±0.23	0.496±0.22	0.494±0.22	0.498±0.21	0.485±0.22	0.480±0.21	0.842±0.22
Total	0.636±0.19	0.636±0.18	0.636±0.18	0.632±0.19	0.639±0.18	0.639±0.18	0.781±0.15

DANN (Domain Adversarial Neural Network, Ganin et al.^24^), IRM (Invariant Risk Minimization, Arjovsky et al.^25^), MetaReg (Meta Learning Regularizer, Balaji et al.^26^), MMD-AAE (Maximum Mean Discrepancy-based Adversarial AutoEncoder, Li et al.^27^), CrossGrad (Cross-Gradient Training, Shankar et al.^28^), LFR (Learn From Randomness, Sui et al.^29^).

We also conducted experiments to evaluate whether incorporating pretrained gene embeddings, derived from a large compendium of single-cell sequencing datasets, could enhance the transfer learning performance of the IC2Bert model. As shown in Figure S6 and Table S4, replacing IC2Bert’s original gene embedding module with scGPT’s pretrained embeddings led to a slight improvement in overall performance. However, the gain appears to be marginal. This modest improvement may be attributed to the architectural and input differences: IC2Bert operates on a preselected set of features, whereas the scGPT model is trained on genome-wide expression data. Thus, the impact of the embedding replacement may be limited in this context. These results suggest that pretrained gene embeddings could offer greater benefits when paired with an appropriate model architecture and under suitable data conditions.

### Effect of training sample size on IC2Bert’s transfer learning

**Fig. 5:**
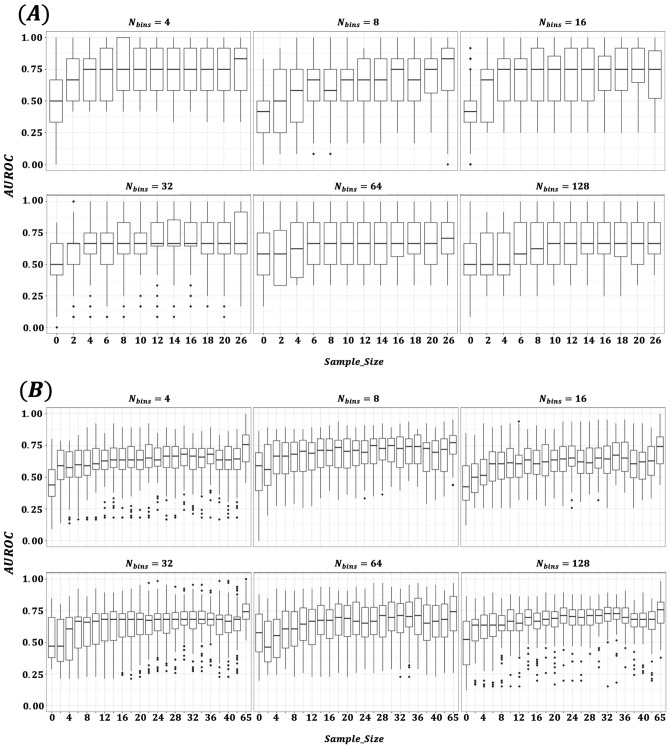
Effect of training sample sizes in performance. (**A**) Miao2018, (**B**) Atezo+Bev_McDermott2018 dataset.

We investigated how the number of balanced training samples influenced IC2Bert’s fine-tuning performance. Figure 5 illustrates the relationship between AUC and training sample size for two representative cohorts, Miao2018 (Fig. 5A) and Atezo+Bev_McDermott2018 (Fig. 5B) datasets. As the number of training samples increased, performance improved substantially, even when starting from a minimal set ($N_{train}=2$). Notably, IC2Bert outperformed zero-shot predictions ($N_{train}=0$), demonstrating that it retained substantial predictive capability from its pretrained representations. In rare cases, zero-shot predictions approached the performance of models trained with one or a few samples, suggesting intrinsic robustness. Additional sample size analyses are provided in the supplementary figures (Figure S1 to S4), which revealed consistent trends across all cohort datasets. Overall, these results highlight IC2Bert’s scalability and adaptability. With additional training samples, the model more effectively fine-tunes its representations to the target dataset, steadily boosting AUC and reinforcing its potential utility in diverse clinical scenarios.

### Feature importance analysis

To gain insight into the genes that most influenced IC2Bert’s predictions, we integrated two complementary methods of feature importance assessment: attention-based and permutation-based analyses. The attention-based approach reveals which genes the model inherently focuses on, while the permutation-based method quantifies the impact of each gene on predictive performance. Combining these approaches helps offset the biases each method may introduce, resulting in a more balanced and reliable measure of gene importance. Table 7 presents the top 10 genes with the highest combined importance scores under a 4-bin discretization scheme, which showed best mean AUC in downstream ICB response prediction task. Each gene’s ranking is accompanied by its frequency of appearance in the top 100, 50, 30, and 10 most important features across 13 holdout datasets, as well as the normalized average importance score. Several of these genes, such as FCRL5, CD19, POU2AF1, and CD79A, are closely associated with B-cell development and function, while others–including CKS1B, TOP2A, PRC1, RRM2, MKI67, and NUSAP1–are well-known markers of cell cycle progression and proliferation.

**Table 7:** Top 10 important features across 13 holdout datasets ($N_{bins}=4$).

Genes	$N_{datasets}$ $^a$				Importance Score$^b$
	Top 100	Top 50	Top 30	Top 10	
CDH1	8	4	3	2	$0.114 \pm 0.139$
RRM2	7	2	0	0	$0.120 \pm 0.094$
NUSAP1	6	2	1	1	$0.120 \pm 0.106$
POU2AF1	6	2	1	0	$0.119 \pm 0.093$
MKI67	5	4	1	1	$0.121 \pm 0.091$
MS4A1	5	3	1	0	$0.115 \pm 0.089$
TOP2A	5	1	0	0	$0.115 \pm 0.087$
FCRL5	4	3	1	1	$0.120 \pm 0.105$
PRC1	4	2	2	2	$0.123 \pm 0.097$
CD79A	3	0	0	0	$0.127 \pm 0.090$

^a^The number of datasets in which genes are included in the Top 100/50/30/10 lists. ^b^The normalized and averaged importance score (± standard deviation).

## Discussion

### Advancing predictive robustness through masked pretraining and fine-tuning

In this study, we introduced IC2Bert, a BERT-like transformer model that employs masked gene expression pretraining and domain-specific supervised fine-tuning to predict immune checkpoint blockade (ICB) responses from heterogeneous RNA-seq datasets. Using a broad range of ICB response cohorts and a Leave-One-Dataset-Out Cross-Validation (LODOCV) framework, IC2Bert demonstrated strong generalizability. Achieving a mean AUROC of 0.781 ± 0.135, it significantly outperformed DNN baselines ($\sim$0.62 AUROC)^30^, Scale Invariant Neural network Classifier (SINC) ($\sim$0.62 AUROC)^23^, gene set-based predictors ($\sim$0.64 AUROC), and advanced domain generalization approaches ($\sim$0.62 AUROC), as shown in Figure S7. This superior performance primarily arises from combining masked pretraining with domain-specific fine-tuning, enabling the model to learn intricate, dataset-agnostic gene relationships without a heavy reliance on labeled data.

To evaluate whether large-cohort foundational model-style pretraining improves generalization, we pretrained IC2Bert on The Cancer Genome Atlas (TCGA) dataset and tested its zero-shot prediction performance on ICB cohorts. The TCGA-pretrained model achieved an average AUROC of 0.512 ± 0.192, which is slightly lower than the model pretrained directly on ICB datasets (0.518 ± 0.200). While transfer learning from the TCGA-pretrained model yielded performance comparable to ICB-based pretraining, these findings indicate that foundational pretraining alone does not guarantee improved predictive power in the context of specific ICB response cohorts. Thus, domain-specific fine-tuning remains essential.

As shown in Figure S5 and Table S3, supplementing masked pretraining with a supervised loss can enhance zero-shot prediction but may reduce subsequent fine-tuning adaptability–highlighting the advantages of the unsupervised masked pretraining approach. A key insight from our ablation study was that using fewer bins (e.g., four) for tokenization improved downstream transfer learning performance. Furthermore, the need for domain-specific fine-tuning was underscored by the limitations of zero-shot predictions, illustrating the importance of adapting learned representations to individual patient cohorts. Taken together, these findings emphasize how IC2Bert’s methodology–drawing on principles from foundational bulk RNA-seq^31^ and scRNA-seq^32,33^ models–captures complex gene expression patterns while preserving the flexibility required to accommodate the unique characteristics of diverse patient populations.

### Adaptability of IC2Bert in transfer learning scenarios

IC2Bert’s adaptability is particularly evident when examining how varying the number of fine-tuning samples affects model performance (Fig. 5). Even with a minimal number of training samples, IC2Bert rapidly approached performance levels comparable to those achieved with substantially larger sample sizes. This suggests that the pretrained representations possess strong predictive power and require only a small amount of domain-specific data to effectively calibrate the model’s outputs to the target domain. As additional samples were introduced, performance gains became modest and stabilized, further indicating that IC2Bert efficiently assimilates domain-specific information without losing its core understanding of gene expression patterns. Such scalability is critical in real-world clinical settings, where data availability can be both limited and heterogeneous. By maintaining strong performance across a broad range of sample sizes, IC2Bert emerges as a highly flexible tool, well-suited for incremental updates as new data become available. This robustness positions the model for practical use in dynamic clinical environments, accommodating varying patient populations, data quality, and cohort sizes, and ultimately improving its utility in personalized treatment planning.

### Biological insights and clinical implications

The feature importance analysis (Table 7) provides a detailed view of the genes most influential in shaping ICB therapy outcomes. Several of the top-ranked genes–MS4A1 (CD20), FCRL5 and CD79A–are closely associated with B-cell functions^34–36^, suggesting that variations in humoral and antigen-presenting activity may critically modulate anti-tumor immunity. At the same time, proliferative markers such as MKI67, TOP2A, PRC1 and RRM2 underscore the importance of cell-cycle dynamics not only in tumor growth but also potentially within rapidly expanding immune subsets that contribute to treatment efficacy^37–40^. Notably, CDH1–an adhesion molecule governing epithelial integrity–appears prominently, further highlighting the interplay between tumor architecture, immune infiltration and therapeutic responsiveness^41^. The appearance of POU2AF1, a transcriptional co-activator in B-cell development, suggests that the differentiation state of lymphoid populations could also influence patient outcomes^42^. By integrating these immunological, proliferative and structural hallmarks, IC2Bert offers a multifaceted portrait of the molecular processes underlying ICB responsiveness.

To validate whether these gene-level signatures reflect differences in immune-cell composition, we applied CODEFACS^43^ to deconvolute bulk RNA-seq data into estimated cell fractions (Figure S8). Consistent with the model’s feature ranking, responders exhibited significantly higher proportions of canonical effector populations–CD8 T cells, CD4 T cells and M1-polarized macrophages–than non-responders. Interestingly, however, CODEFACS did not detect a statistically significant difference in overall B-cell abundance between the two groups. This apparent discrepancy may arise because IC2Bert captures B-cell functional states or specific subpopulations–such as antibody-secreting or tertiary lymphoid structure-associated B cells–that bulk deconvolution cannot distinguish. It may also reflect context-dependent roles of B cells, where spatial organization and activation status, rather than total numbers, drive therapeutic benefit.

From a clinical standpoint, these insights can refine patient stratification and inform treatment decisions. Oncologists may leverage the combined gene-level and deconvolution metrics to identify individuals most likely to benefit from ICB therapy, guiding more personalized and effective strategies. Ultimately, by linking complex genomic patterns to actionable cellular phenotypes, IC2Bert advances the goal of precision oncology.

### Limitations, future directions, and multimodal integration

While our results are promising, certain limitations persist. Although the aggregated dataset spans 13 cohorts, some cancer types remain underrepresented, and increasing both the size and heterogeneity of training cohorts will be essential to bolster IC2Bert’s generalizability. Clinically, bulk RNA-seq remains attractive thanks to well-established workflows, relative cost-effectiveness, and the ability to mine large retrospective collections for model development and validation. However, by averaging expression across all cells, bulk profiles obscure rare or spatially confined populations and eliminate information about cell–cell interactions. Deconvolution approaches such as CODEFACS can partially reconstruct cell-type abundances but remain sensitive to tumor purity, stromal admixture and the choice of reference signatures, which may limit their precision in individual patient settings.

Single-cell and spatial transcriptomic technologies address many of these shortcomings by directly profiling cellular heterogeneity, cell states and tissue organization. These modalities can uncover key microanatomical features–such as exhausted T-cell niches or tertiary lymphoid structures–that are invisible to bulk assays yet highly relevant to ICB efficacy. Nevertheless, widespread clinical adoption of single-cell and spatial methods is currently hindered by higher per-sample costs, complex tissue requirements, specialized library preparations and nonstandardized analytical pipelines. To reconcile breadth and depth, future studies should pursue multimodal integration, combining bulk RNA-seq with orthogonal data types–genomic mutations, proteomic signatures or radiographic imaging–to derive richer, more robust predictors of therapeutic response.

Finally, interpretability remains an important challenge. Although IC2Bert identifies biologically plausible features, the underlying decision pathways are not fully transparent. Future work will focus on pretraining IC2Bert components on large single-cell or spatial atlases and applying advanced explainable-AI techniques to deconvolute latent model reasoning. By illuminating how specific cell states, spatial contexts and molecular alterations drive predictions–and by uniting multiple data modalities–we can enhance both the mechanistic insight and the clinical utility of ICB response models, ultimately advancing more precise, personalized oncology interventions.

## Conclusions

IC2Bert marks a notable advancement in immunotherapy response prediction by seamlessly integrating masked gene expression pretraining with domain-specific supervised fine-tuning. This approach effectively tackles the challenges posed by cohort heterogeneity and demonstrates robust, generalizable performance across diverse datasets. By relying solely on widely available bulk RNA-seq data, IC2Bert achieves high predictive accuracy and adaptability, making it a promising tool for personalized oncology. The publicly available IC2Bert model and source code set the stage for ongoing research and enhancement. Future directions include expanding the range of datasets, incorporating additional data modalities, and employing advanced interpretability techniques. We anticipate that these efforts will further elevate IC2Bert’s clinical utility, ultimately guiding more precise therapy selection and improving patient outcomes in the rapidly evolving landscape of personalized cancer treatment.

## Methods

### Datasets

We employed 13 immune checkpoint blockade (ICB) response cohorts derived from 11 published studies, encompassing a total of 1,214 patients (31.8% responders). Metastatic melanoma was the most frequently studied cancer type, while other cancer types were represented either individually or in a single pan-cancer study. Most patients received anti-PD-1 or anti-PD-L1 monotherapy, with a smaller subset treated using combinations of PD-1/PD-L1 and CTLA-4 inhibitors or bevacizumab. The ICB+bevacizumab cohort was considered distinct from the ICB monotherapy cohorts, even if derived from the same study. Table 1 provides detailed patient statistics for all 13 ICB response cohorts.

### Feature selection

Due to the high dimensionality of gene expression datasets and the relatively small number of samples in each domain-specific dataset, reducing the feature space is often beneficial when employing machine learning (ML) or deep learning (DL) approaches for ICB response prediction. This reduction helps to decrease model complexity and training time. In this study, we curated 1,179 genes from 34 published gene sets associated with immune checkpoint blockade (ICB) response prediction, which have been identified as predictors of ICB response in certain datasets (Supplementary Table 1). Of these, 983 genes were consistently measured across all 13 ICB cohort datasets, and we used these 983 genes as input features for the IC2Bert model.

### Model architecture

The IC2Bert model is an encoder-only transformer architecture specifically designed for processing gene expression data and tailored for immunotherapy response prediction. It begins by transforming continuous gene expression values into discretized tokens through a tokenization process, making them suitable for input into a transformer model. Each input token represents a particular gene and its corresponding expression bin, which are then processed through two primary embedding layers: an expression embedding layer that maps discretized expression tokens into 128-dimensional vectors and a gene embedding layer that maps 983 gene identifiers into 64-dimensional vectors. To align the gene embeddings with the expression embeddings, a gene projection layer projects the 64-dimensional gene embeddings to 128 dimensions using a linear transformation. The combined embeddings, capturing both biological context and discretized expression levels, are then fed into four self-attention blocks. Each block comprises a layer normalization, a multi-head self-attention mechanism with query, key, and value projections, an output linear layer, followed by another layer normalization and a feed-forward network. Dropout and layer normalization are applied throughout for regularization and stability. After processing through the self-attention blocks, the model branches into two heads: an MLM head that predicts masked tokens by mapping the 128-dimensional representations to $N_{bins} + 1$ classes, corresponding to the discretized expression bins plus a special token (i.e., <mask>); and a simple classification head that predicts the binary immunotherapy response label by reducing the 128-dimensional pooled representation to a single logit. The <mask> token is a learned embedding that allows the model to learn contextual relationships during masked language modeling, where selected expression tokens are replaced with <mask> during pretraining and the model is trained to recover the original expression bin.

### Gene expression tokenization

We used log-transformed expression values, $log(TPM+1)$, as input for the tokenization process. To prepare the data for transformer-based modeling, continuous gene expression values were discretized into tokens using a quantile-based tokenizer. For each gene within a dataset, the tokenizer computed bin thresholds from the training subset and then applied the same thresholds to the corresponding test subset, ensuring consistent and unbiased discretization. The tokenizer partitions the range of expression values into a predefined number of bins (e.g., 4, 8, 16, 32, 64, or 128), assigning each expression value to its corresponding bin. Each bin represents a discrete token that the model learns to interpret. This transformation enables the model to identify categorical patterns in gene activity while accommodating expression-level variability across samples. To examine the impact of bin granularity on predictive performance, we performed an ablation study across six bin sizes: 4, 8, 16, 32, 64, and 128. In addition, we adopted a hybrid approach in which each gene is assigned a unique learnable embedding vector to represent its identity, allowing the model to distinguish between genes independent of their expression values. These gene identity embeddings are projected into the same dimensional space as the expression embeddings and then combined via element-wise addition. This fused representation enables the model to jointly capture gene-specific and expression-level information in a unified input format.

### Pretraining

Pretraining involves a masked language modeling (MLM) objective, where random tokens in the input are replaced with a special mask token or retained unchanged according to predefined probabilities. The model is then trained to recover the original values of these masked tokens using a cross-entropy loss function that focuses exclusively on the masked positions. This process enables the IC2Bert model to learn meaningful and context-aware representations of gene expression, capturing complex patterns and relationships in the underlying biological data.

To evaluate the effect of pretraining on different datasets, each pretraining run was initialized independently using random parameter initialization. No weights were shared or transferred across runs. Specifically, for each evaluation, the model was pretrained from scratch on the pretrain subsets of 12 datasets (excluding the designated holdout) using the MLM objective. This design ensures that the performance reflects the contribution of each dataset combination, without the influence of cumulative training effects or prior knowledge from other runs.

### Transfer learning

For downstream tasks, the pretrained IC2Bert model is fine-tuned on the training dataset of the test domain. During fine-tuning, the model’s parameters are adjusted to optimize binary cross-entropy or focal loss, depending on dataset-specific configurations. Hyperparameters such as batch size, learning rate, and dropout rate are dynamically adjusted based on dataset size and class imbalance.

To determine and refine optimal hyperparameters for each dataset, we employed a data-driven, context-sensitive approach informed by dataset size, class distribution, and cancer type. We began with baseline configurations grouped into small, medium, or large dataset tiers, then introduced adjustments in response to observed class imbalance, data heterogeneity, and domain-specific factors. For instance, smaller or heavily imbalanced datasets prompted the use of focal loss and extended patience values, whereas larger datasets benefited from strategies like gradient centralization and mild regularization.

Focal loss parameters were dynamically set to tailor gamma ($\gamma$) and alpha ($\alpha$) values based on class ratios. Dataset-specific hyperparameters were tuned for batch size, learning rate, dropout, and related optimization controls. This flexible framework enabled continuous, incremental refinement: initial general-purpose defaults were iteratively adapted as the training process revealed dataset-specific requirements.

To ensure consistency and reproducibility, all selected hyperparameters were documented and stored, facilitating uniform transfer learning protocols across diverse datasets. Throughout training, we monitored performance metrics such as AUC, applying early stopping criteria and checkpointing to prevent overfitting and revert to the most effective model state. By integrating these adaptive and systematic strategies, we enhanced both the stability and accuracy of ICB response predictions across a wide range of clinical datasets.

For the ensemble prediction, we used 12 models–each pretrained on 12 datasets (excluding one) and fine-tuned on the training subset of the held-out dataset. These 12 fine-tuned models were then used to generate ensemble predictions for the test subset of the held-out dataset. Importantly, the test subset was never used during either the pretraining or fine-tuning stages. This setup ensures a fair comparison between the performance of a single fine-tuned model and the ensemble of 12 models on the same held-out test subset.

### Sample size analysis

To evaluate the impact of training sample size on transfer learning performance, we performed a systematic analysis using the holdout datasets. For each holdout dataset, we varied the number of training samples from 2 up to the maximum number of available balanced samples, incrementing in steps of 2 to maintain equal representation of positive (responders) and negative (non-responders) classes. This approach ensures that the class balance does not confound the effects of sample size on model performance. For each sample size, we conducted R=10 independent iterations, randomly selected an equal number of positive and negative samples from the training set. The selected samples were used to fine-tune the pretrained IC2Bert model. The performance of the fine-tuned models was evaluated on a separate test set composed of samples not used during fine-tuning. The primary metric for assessing model performance was the Area Under the Receiver Operating Characteristic Curve (AUC). For each sample size and iteration, the model’s AUC was calculated on the test set predictions.

### Feature importance analysis

To identify the genes most influential in our model’s predictions, we conducted a feature importance analysis using two complementary methods: attention-based importance and permutation-based importance. Attention-Based Importance leverages the attention mechanisms inherent in transformer models. Specifically, we extracted the attention weights from each of the model’s layers and heads and normalized as shown below.

$$I_i^{attent}=\frac{|w_i^{attent}|}{(\sum _j|w_j^{attent}| )}$$

where i represents gene of interest and j represents all genes in the feature space. The attention scores reflect how much the model focuses on each gene when making predictions. Higher attention scores indicate greater influence of a gene on the model’s internal representation. Permutation-Based Importance assesses the impact of each gene on the model’s predictive performance. For each gene, we permuted its expression values across the dataset, effectively disrupting any relationship between that gene and the target variable. We then measured the decrease in model performance, quantified by the drop in the area under the receiver operating characteristic curve (AUC), as shown below.

$$I_i^{perm}=\frac{1}{R} {\sum _{r=1}^R}(AUC_{baseline}-AUC_{perm(i, r)}), \;\;\; I_i^{perm}=\frac{|I_i^{perm}|}{\sum _j|I_j^{perm}|}$$

where i represents gene of interest, r represents number of repetitions of the permutation and j represents all genes in the feature space. Genes whose permutation leads to a significant performance drop are considered more important. To obtain a robust measure of feature importance, we combined the attention and permutation scores, as shown below.

$$I_i^{comb}=\alpha I_i^{attent}+(1-\alpha )I_i^{perm}$$

We assigned a weight of 40% to the attention-based importance and 60% to the permutation-based importance, reflecting the empirical influence on model performance. The combined score provides a balanced view of each gene’s significance, considering both the model’s internal focus and the practical impact on predictions.

### Implementation details

IC2Bert is implemented in Python 3.10 and builds on the JAX ecosystem–using JAX v0.4.34 and Haiku v0.0.13 to define the transformer backbone, Optax v0.1.7 for optimization (AdamW with warmup-cosine schedules and gradient clipping), and Orbax v0.7.0 for checkpoint management. Data preprocessing leverages pandas v2.2.3 and numpy v1.24.3 for I/O and array operations, scikit-learn v1.5.2 for stratified splits, and a custom BinnedExpressionTokenizer for quantile-based binning. Progress and parallel analyses use tqdm v4.66.5 and joblib v1.4.2, while matplotlib v3.9.2 and seaborn v0.13.2 support visualization. All dependencies were installed via pip v24.2 in a Linux environment.

### Hyperparameter selection

We used a sample-size–dependent hyperparameter selection strategy rather than an exhaustive grid search. At startup, each hold-out cohort is classified as “small” ($<50$ samples), “medium” ($50 - 99$) or “large” ($\ge 100$), and this label determines sensible defaults for batch size (8/16/32), base learning rate (5E-5, 1E-4, 2E-4), AdamW weight decay (0.015/0.01/0.008), dropout rate (0.2/0.15/0.1), gradient-clipping norm (0.5/0.75/1.0), warmup–decay schedule and early-stopping patience (20/15/10). This simple, data-aware heuristic proved robust across folds and datasets, delivering consistently strong held-out performance without costly grid searches.

## Supplementary Information

Supplementary Information 1.

Supplementary Information 2.

## Data Availability

• Bulk RNA-seq data are publicly available at GEO: GSE91061, GSE78220; dbGaP: phs001493.v1.p1, phs000452.v2.p1, phs000452.v3.p1, phs002822.v1.p1; ENA: PRJEB25780; and EGPA: EGAS00001002928, EGAS00001003280, EGAS00001002556, EGAD00001008129, EGAD00001008130. Preprocessed datasets (open access only) are available on the CIDE platform^55^ (https://cide.ccr.cancer.gov/). • All original code has been deposited at github (https://github.com/data2intelligence/ic2bert) and is publicly available as of the date of publication. • Any additional information required to reanalyze the data reported in this paper is available from the lead contact upon request.
