# Supplementary material for: IC2Bert: masked gene expression pretraining and supervised fine tuning for robust immune checkpoint blockade (ICB) response prediction
Source: Sci Rep. 2025 Aug 1;15:28044. doi: 10.1038/s41598-025-14166-x (PMC12313928; doi:10.1038/s41598-025-14166-x)
Supplement: Supplementary file 1 — Supplementary Information 1. [file 41598_2025_14166_MOESM1_ESM.pdf]

IC2Bert: Masked Gene Expression Pretraining and Supervised Fine Tuning for Robust Immune Checkpoint Blockade (ICB) Response Prediction

Seongyong Park[1,*], Seonkyu Kim[2], and Peng Jiang[1]

[1]Cancer Data Science Lab, CCR, NCI, NIH, Bethesda, MD 20892, USA
[2]Aging Convergence Research Center, KRIBB, Daejeon 34141, Republic of Korea
[*]Correspondence: seongyong.park@nih.gov

ABSTRACT

Bulk RNA-seq-based prediction of immune checkpoint blockade (ICB) responses has been extensively studied to distinguish responders from non-responders. However, cohort heterogeneity remains a major challenge, hindering the robustness and generalizability of predictive models across diverse RNA-seq datasets. In this study, we present IC2Bert, a novel model that employs masked gene expression pretraining combined with domain-specific supervised fine-tuning to enhance predictive robustness across heterogeneous ICB response cohorts. To ensure an objective evaluation, we assessed the model's performance using a Leave-One-Dataset-Out Cross-Validation (LODOCV) approach. IC2Bert demonstrated significantly improved predictive accuracy and robustness compared to existing methods, effectively addressing the challenges posed by cohort heterogeneity. The IC2Bert model and its source code are publicly available on GitHub: https://github.com/data2intelligence/ic2bert.

SUPPLEMENTAL INFORMATION

Document S1

- Table S1. List of curated ICB response prediction gene sets (Sheet 1).
- Table S2. Top 10 important features across 13 holdout datasets with respect to N_{bins} (Sheet 2).
- Table S3. Performance evaluation results by different pretraining objectives (Sheet 3).
- Table S4. Performance comparison between IC2Bert and IC2Bert with scGPT's gene embedding vectors (Sheet 4).
- Figures S1–S4. Effect of training sample sizes on performance in other datasets (VanAllen2015, Riaz2017, Atezo_McDermott2018, and Ravi2023).
- Figure S5. Comparison between MLM and MLM + BCE loss-based pretraining strategies.
- Figure S6. Comparison between IC2Bert and IC2Bert with scGPT's gene embedding vectors.
- Figure S7. Pairwise performance comparison among all methods in LODOCV setting.
- Figure S8. Comparison of immune cell-type abundances in ICB responders versus non-responders.

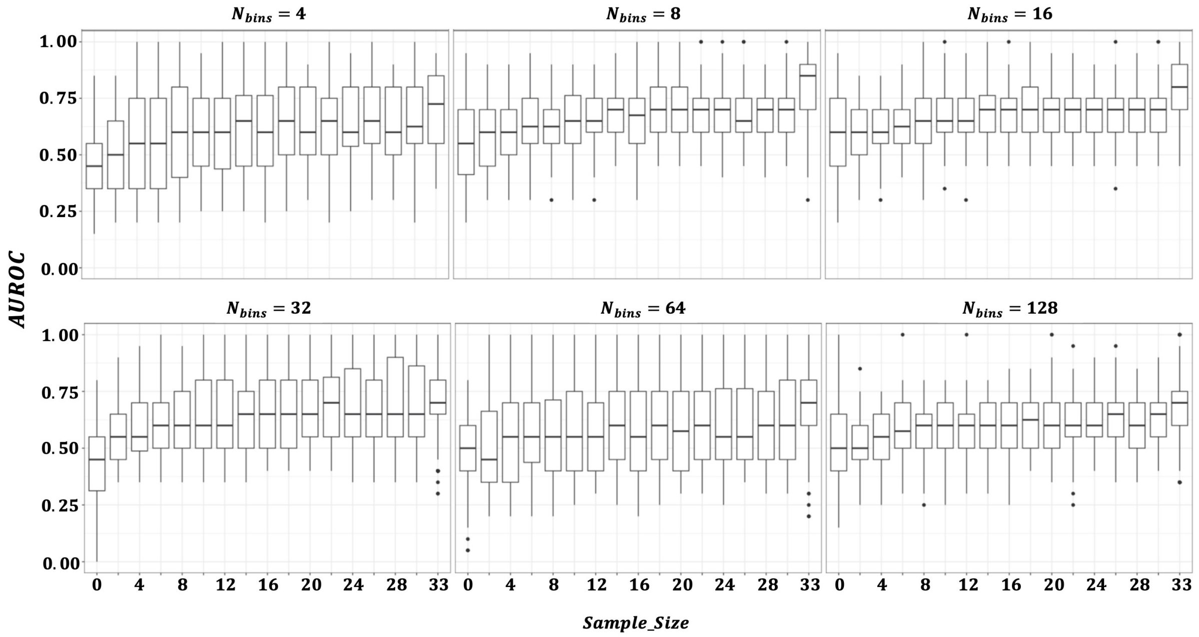

Figure S1. Effect of training sample sizes in performance for VanAllen2015 dataset.

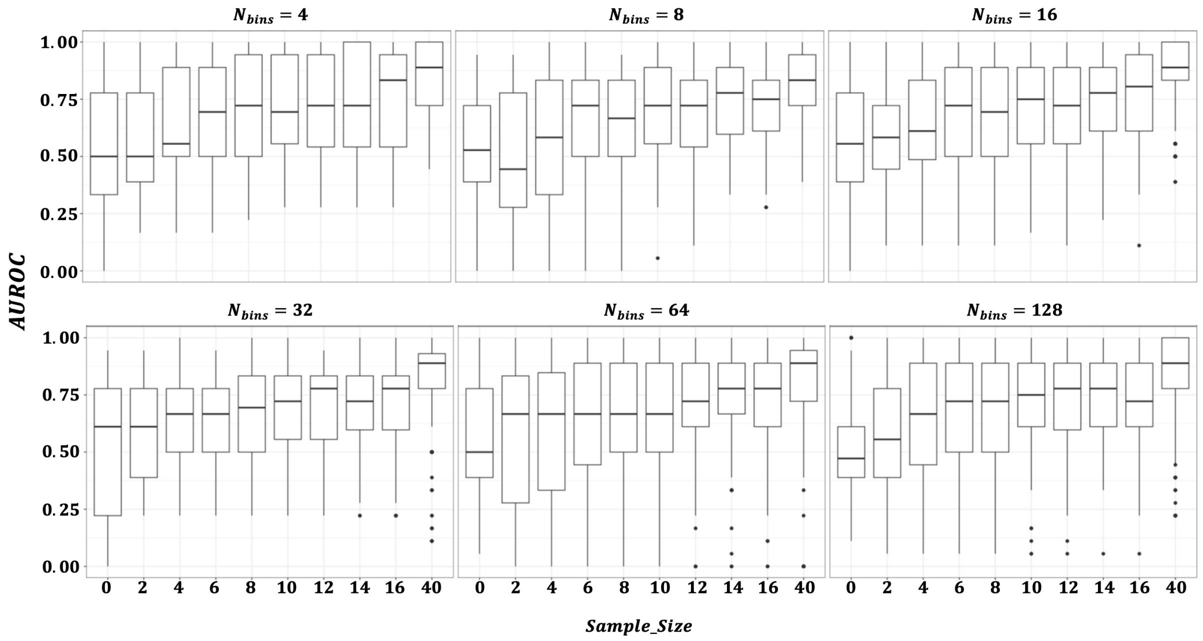

Figure S2. Effect of training sample sizes in performance for Riaz2017 dataset.

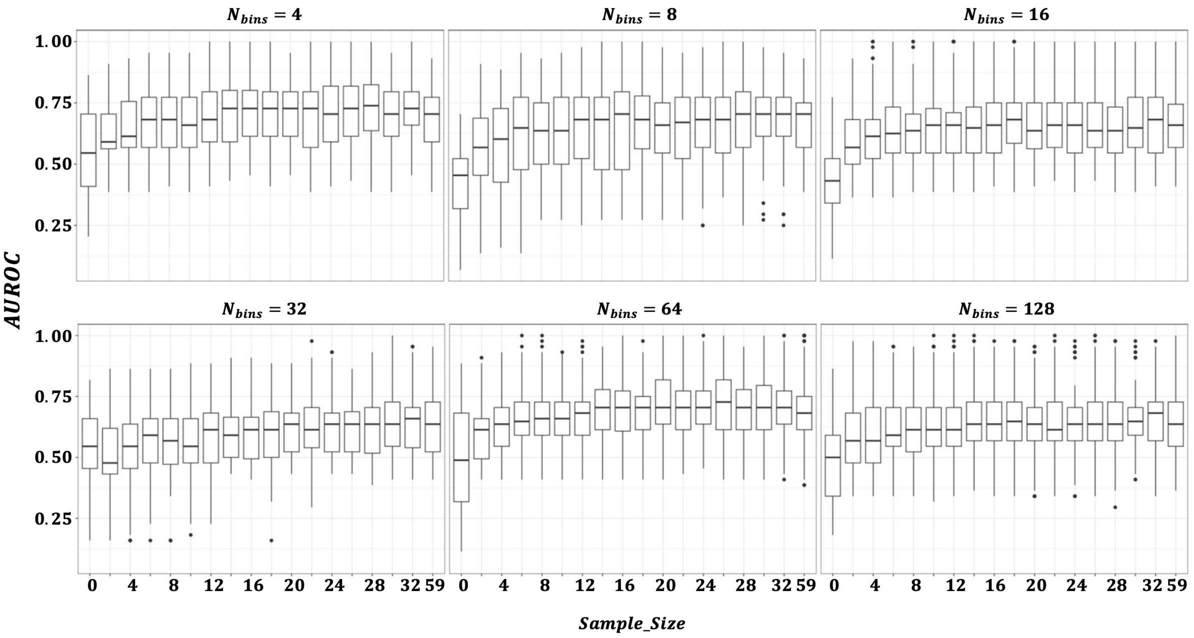

Figure S3. Effect of training sample sizes in performance for Atezo_McDermott2018 dataset.

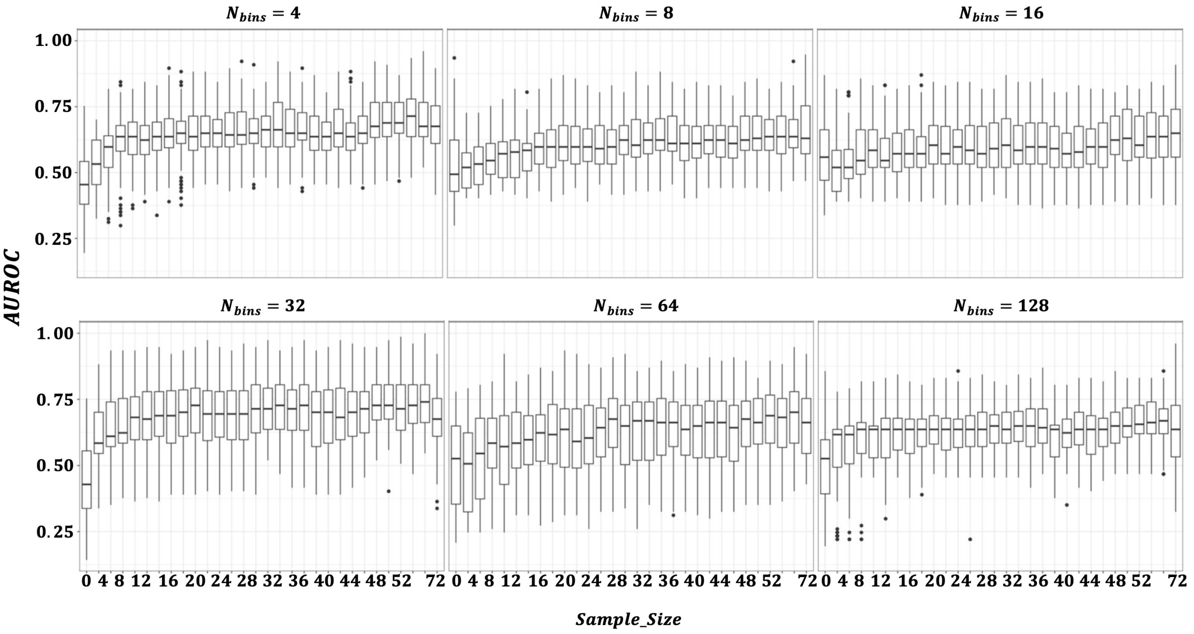

Figure S4. Effect of training sample sizes in performance for Ravi2023 dataset.

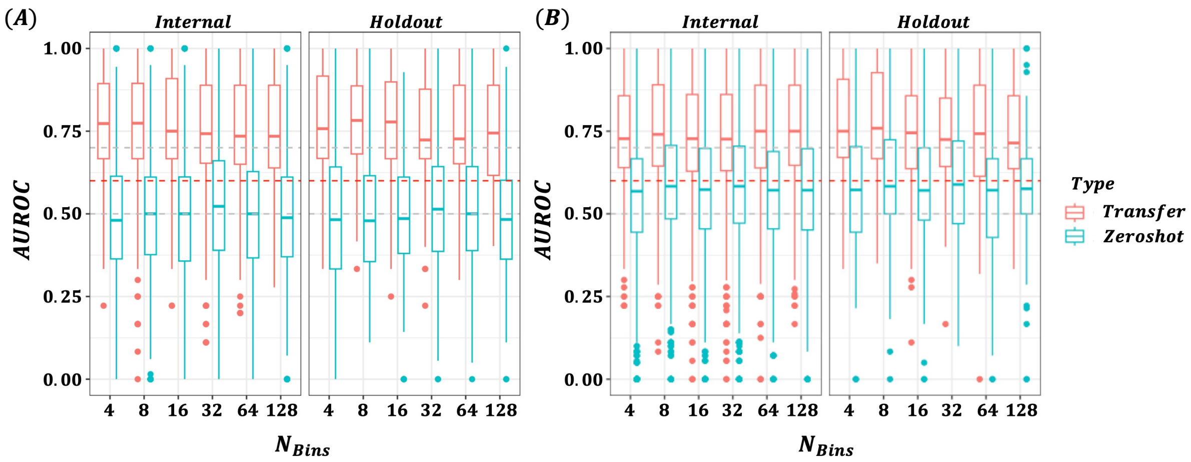

Figure S5. Comparison between MLM and MLM + BCE loss based pretraining strategies (A) MLM loss function (B) MLM+BCE Loss function.

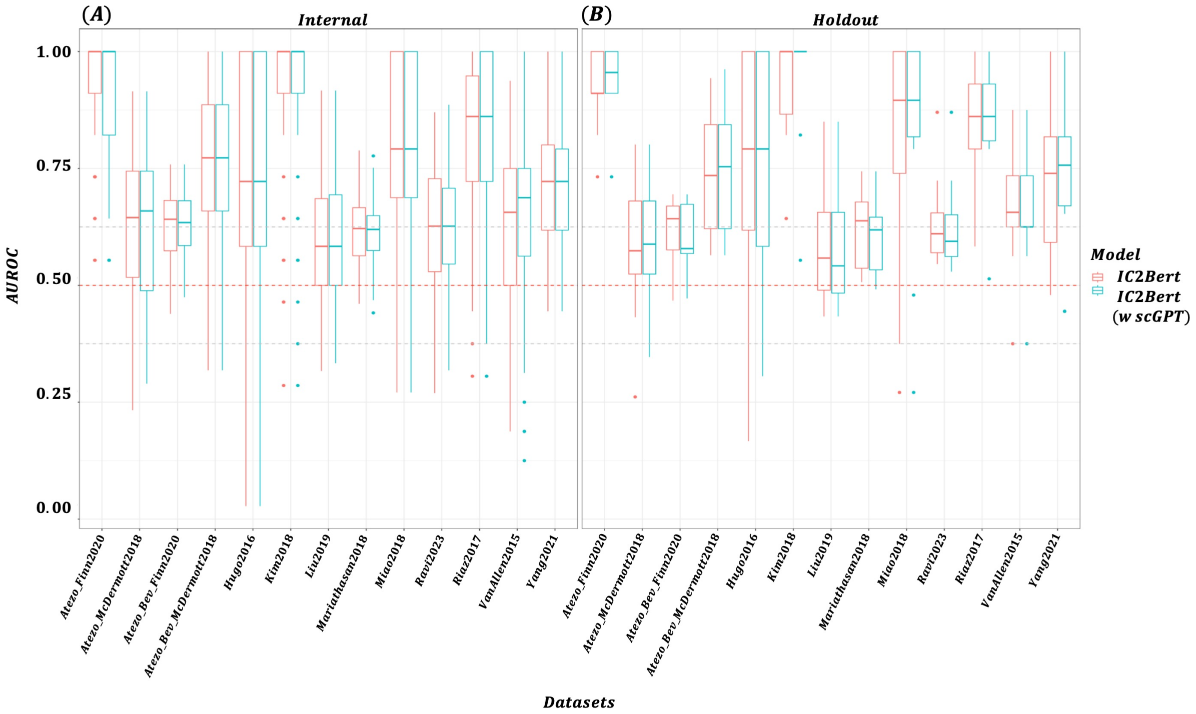

Figure S6. Comparison of performance between IC2Bert and IC2Bert with scGPT's pretrained gene embedding vectors ($N_{bins} = 4$).

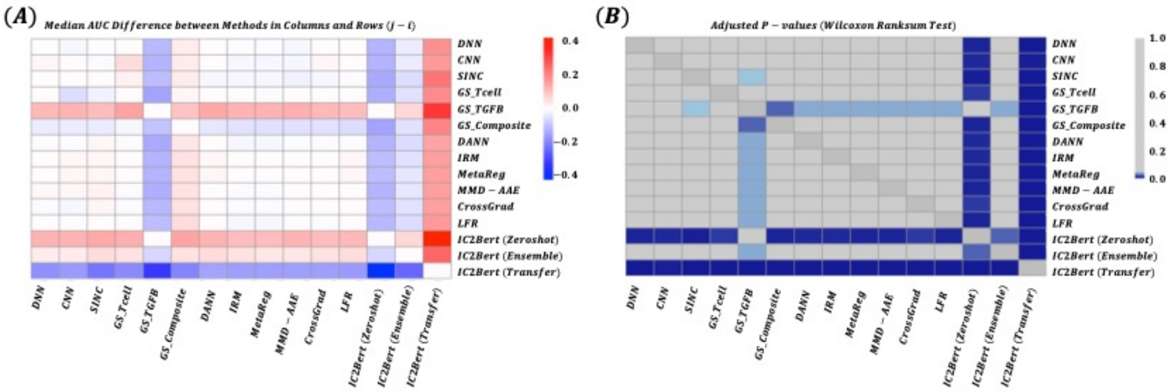

Figure S7. Pairwise performance comparison among all methods in LODOCV setting. (A) Median AUC difference between methods in column j and row i. (B) Adjusted p-values of pairwise comparisons (Wilcoxon rank-sum test), with dark blue indicating $p < 0.05$ and light blue indicating $p < 0.10$.

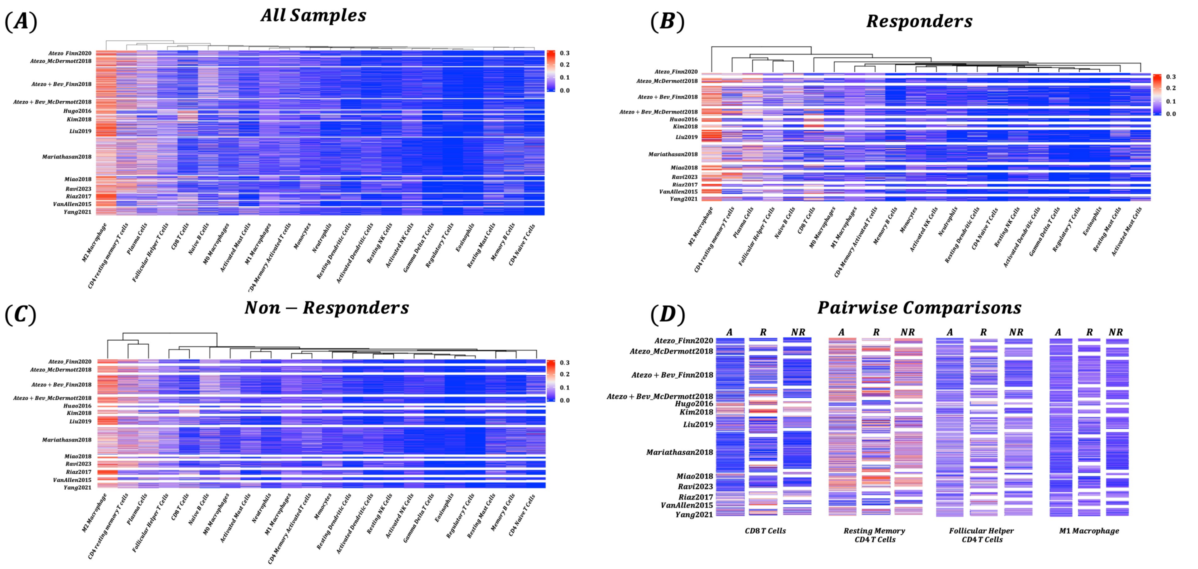

Figure S8. Comparison of immune cell-type abundances in ICB responders versus non-responders. (A) Heatmap showing the relative proportions of all immune cell types across every sample from 13 independent cohorts. (B) Heatmap of cell-type fractions in responder samples only. (C) Heatmap of cell-type fractions in non-responder samples only. (D) Differential abundance analysis between responders and non-responders: four effector cell populations exhibited significant differences (Wilcoxon rank-sum test, Benjamini–Hochberg $FDR < 0.05$).
